# Prediction Models for Decision-Making on Chagas Disease

**DOI:** 10.5935/abc.20170059

**Published:** 2017-05

**Authors:** Fernanda de Souza Nogueira Sardinha Mendes, Pedro Emmanuel Alvarenga Americano do Brasil

**Affiliations:** Instituto Nacional de Infectologia / Fundação Oswaldo Cruz (INI/FIOCRUZ), Rio de Janeiro, RJ - Brazil

**Keywords:** Chagas Disease, Chagas Cardiomyopathy, Decision Support Techniques, Decision Making

To investigate the relationship between future or unknown outcomes and baseline health states among people with specific conditions, prediction models are an interesting strategy used to assist diagnosis, prognosis and treatment.^1^ They estimate the likelihood of clinical events taking into account clinical relevant measures and complementary tests.^2^ These predictors and their importance vary between the different events of interest and their prediction ability varies when considered singly or in combination with other predictors.^2^ They may facilitate simple and direct comparisons of risks, individualize treatment regimens, and may refine prognosis stratification of patients, especially when many prognostic factors are known. Models have to be simple, easy to use and lead the clinician to make decisions which are more likely to bring benefit to the patients.

The World Health Organization estimates that 7-8 million people worldwide are infected with T.cruzi.^3^ Chagas disease is endemic in Latin America and the immigration pattern in the past years has made this disease an important health issue in many countries. In United States, more than 300,000 individuals might be infected^4^ and one study estimated that 3.5% of immigrants to Canada from Latin America were infected.^5^ Physicians should be able to recognize signs and symptoms of Chagas disease as globalization increase the burden of this disease in non-Latin American countries, where vector transmission is unlikely to occur.^5^

Chagas disease has a chronic and persistent inflammation of the myocardium that leads to destruction of cardiomyocytes, arrhythmias, and embolic events, which are the leading causes of death. The intensity and aggression of Chagas disease differ substantially from that observed in other cardiomyopathies and these factors are responsible for the worse prognosis.^6^ Because of its unique clinical and pathological characteristics a decision making based on other cardiomyopathies parameters might not offer all the potential benefit to patients, therefore improvements/adaptations on this knowledge are required. However, in the field of Chagas disease, there are few risk prediction models to assist decision making.

Here, we provide comments and a brief discussion regarding the prediction models on Chagas disease field currently available in the literature. In 2016, Brasil et.al.^7^ developed and validated a diagnostic decision support tool to decide about proceed or not to diagnostic investigations for chronic Chagas disease. The following predictors were identified: sex, age, referral from blood bank, history of living in a rural area, recognizing the kissing bug from pictures, systemic hypertension, number of siblings with Chagas disease, number of relatives with history of stroke, electrocardiogram with low voltage, anterior superior divisional block, pathologic Q wave, right bundle branch block, and extrasystoles. This model was developed and temporally validated in a single center study, with very good discrimination and calibration performance in both samples. Therefore it could be recommended in ordinary use in diagnostic investigation despite its impact is not yet known.

The second model in discussion is the one to predict severe or moderate systolic dysfunction in Chagas disease.^8^ It was used based on the following clinical, electrocardiographic and radiologic data: sex, chest X-ray, right bundle branch block, anterior superior divisional block, ventricular extra systole, pathologic Q-wave, primarily ventricular repolarization alterations, left bundle branch block, and pacemaker rhythm. The validation was in a rural cohort of patients with Chagas disease, randomly selected and submitted to clinical, electrocardiogram and echocardiographic evaluation. A normal electrocardiogram excluded the presence of moderate or severe dysfunction, not requiring the application of statistical models in 43% of this population. This tool can be widely used, including rural areas, since it needs simple clinical, electrocardiographic and radiological data. It can provide the decision to start specific treatment to heart failure until echocardiography is not available, identifying patients who may have benefit from this early treatment. It was validated in a fully independent sample, and it had good performance in both cohorts. Thus it can be recommended for ordinary use in urban and rural populations.

In 2006, Rassi et.al.^9^ developed and validated a risk score for predicting overall death in Chagas' heart disease. It was found that six clinical features were important in predicting death: NYHA class III or IV, cardiomegaly on chest radiography, segmental or global wall-motion abnormalities on echocardiography, nonsustained ventricular tachycardia on Holter monitoring, low QRS voltage on electrocardiography, and male sex. This model was developed and validated in a fully independent concurrent cohort and its performance in both cohorts was good. However, this model needs several complementary tests to estimate individual risk (e.g. Holter monitoring), and it does not evaluate the left ventricle ejection fraction, a known strong prognostic predictor in Chagas heart disease.^10^ Additionally, it is difficult to make decision from its estimates as Chagas disease has three main death mechanisms that require completely different treatment approaches (i.e. stroke, sudden death, and heart failure).

Another interesting model studied the risk of sudden death in chronic Chagas' heart disease.^11^ Four independent predictors were identified, each of which was assigned a number of points proportional to its regression coefficients: QT-interval dispersion, syncope, ventricular extrasystoles and severe dysfunction of the left ventricle. The risk scores for each patient were divided in three groups: low risk, intermediate, and high risk. This study showed that a simple model can predict sudden death with a good clinical relevance of the model with C statistic score of 0.84. Highlighting Chagas disease unique characteristics, the QT-interval dispersion was the strongest predictor of sudden-death in Chagas heart disease, which is not common in other etiologies. Unfortunately, this model was not yet been externally validated, and it requires QT-dispersion, which is not easily measured. Therefore it cannot be recommended for ordinary practice and its applicability depends on the setting. Another research group conducted the "SEARCH-RIO study" that evaluated electrocardiogram, signal-averaged electrocardiogram, and Holter monitoring variables in chronic Chagas disease as predictors of cardiac death and new onset ventricular tachycardia as a composite outcome.^12^ This long term follow-up developed a risk stratification score showing that electrical markers are independent predictors of adverse outcome. The electrical markers were: abnormal Q-wave, previous ventricular tachycardia episodes, 24-h standard deviation of normal RR intervals < 100 ms, and positive intraventricular electrical transients on signal-averaged electrocardiogram. The study had a good relevance with C-statistic of 0.89, but was not externally validated. Additionally, the model's composite outcome makes decision more complicated, and the requirement of Holter monitoring makes its applicability setting dependent. Therefore it cannot be recommended for ordinary practice.

Sousa et.al.^13^ studied the risk and benefit of prevention strategies of cardioembolic ischemic stroke in Chagas disease. The factors that increased the risk of an event were: systolic dysfunction, apical aneurysm, primary alteration of ventricular repolarization and age > 48 years. Based on the analysis, four risk groups were defined to the rate of events in these groups. The suggestion is to use of warfarin for high-risk patients (score 4 or 5), acetylsalicylic acid or warfarin for those with moderate risk (score 3), acetylsalicylic acid or no intervention for the low risk group (score 2) and no prophylaxis for the very low risk group (score 0 or 1). This model was developed in a very large sample, and it has a very good performance. However, with the availability of new anticoagulants, the applicability of this model is setting dependent. Additionally, it was not yet validated externally, thus cannot be recommended for ordinary practice.

Benznidazole is the main trypanocidal drug used to treat Chagas disease. This drug is recommended (Class I) as trypanocidal treatment in the acute phase of Chagas disease, congenital Chagas disease, chronic phase of Chagas disease in children aged ≤ 12 years, organ donor with Chagas disease, and reactivation antiparasitic treatment in coinfection Chagas/HIV.^10^ More than 30% of patients treated may present adverse drug reactions.^14^ There is a prediction model to identify patients with high risk to develop adverse reactions to benznidazole and to identify the risk of requiring benznidazole interruption due to adverse reactions.^15^ It was found that female sex, graduation from elementary school, and white and mulatto races were considered to predict overall adverse drug reactions and treatment discontinuation. This model was developed in a small sample; it has a moderate discrimination and a good calibration performance. However, it was not yet externally validated.

The use of clinical prediction models can be an interesting strategy to assist diagnosis, prognosis and treatment decision making. However, the user must be concerned with the applicability of the model for the patient under care. All the mentioned models were developed in urban cohorts; therefore these samples resemble in many aspects the populations of migrants with Chagas disease in non-endemic countries. Even if some models presented are not validated and cannot be widely used, they raise a consciousness of which clinical aspects health-care providers should concern with when assessing patients with Chagas disease. Nevertheless, the correct interpretation and application of Chagas disease prediction models remains a challenge to clinical decision-making. To fulfill the purpose of facilitating these models use, we turn available online calculators concerning the prediction models in the following link: http://shiny.ipec.fiocruz.br:3838/pedrobrasil/. It´s important to remind that this website for the calculation of risk prediction scores are not intended to replace the currently available guidelines for chronic Chagas disease health care, instead they are intended to complement, facilitate the application of current recommendations, improving medical decision making and ultimately bring more benefit to patients with Chagas disease.

Updating the existing models and providing new ones can be useful for several purposes in the field of Chagas disease. This raises the question of models that are likely to bring benefit, such as: how to predict the progression of indeterminate form for cardiac and digestive forms, models for cardiac transplant indication; one that can predict which patient would have benefit with benznidazole treatment; and a model in the field of cardiac rehabilitation to predict who will have benefits. In light of the personalized medicine era, further research is needed to reach individual predictions, where genetic or innate biomarkers can play bigger roles, as well as making these prediction instruments friendlier.
